# Improvements in treatment planning calculations motivated by tightening IMRT QA tolerances

**DOI:** 10.1002/acm2.12524

**Published:** 2018-12-31

**Authors:** Cassandra Stambaugh, Justin Gagneur, Arielle Uejo, Edward Clouser, Gary Ezzell

**Affiliations:** ^1^ Department of Radiation Oncology Tufts Medical Center Boston MA 02111 USA; ^2^ Department of Radiation Oncology Mayo Clinic in Arizona Phoenix AZ 85054 USA; ^3^ Department of Radiation Oncology Karmanos cancer Center at McLaren Flint Flint MI 48532 USA

**Keywords:** IMRT QA, patient‐specific QA, treatment planning systems

## Abstract

Implementing tighter intensity modulated radiation therapy (IMRT) quality assurance (QA) tolerances initially resulted in high numbers of marginal or failing QA results and motivated a number of improvements to our calculational processes. This work details those improvements and their effect on results. One hundred eighty IMRT plans analyzed previously were collected and new gamma criteria were applied and compared to the original results. The results were used to obtain an estimate for the number of plans that would require additional dose volume histogram (DVH)‐based analysis and therefore predicted workload increase. For 2 months and 133 plans, the established criteria were continued while the new criteria were applied and tracked in parallel. Because the number of marginal or failing plans far exceeded the predicted levels, a number of calculational elements were investigated: IMRT modeling parameters, calculation grid size, and couch top modeling. After improvements to these elements, the new criteria were clinically implemented and the frequency of passing, questionable, and failing plans measured for the subsequent 15 months and 674 plans. The retrospective analysis of selected IMRT QA results demonstrated that 75% of plans should pass, while 19% of IMRT QA plans would need DVH‐based analysis and an additional 6% would fail. However, after applying the tighter criteria for 2 months, the distribution of plans was significantly different from prediction with questionable or failing plans reaching 47%. After investigating and improving several elements of the IMRT calculation processes, the frequency of questionable plans was reduced to 11% and that of failing plans to less than 1%. Tighter IMRT QA tolerances revealed the need to improve several elements of our plan calculations. As a consequence, the accuracy of our plans have improved, and the frequency of finding marginal or failing IMRT QA results, remains within our practical ability to respond.

## 1 INTRODUCTION

The clinical relevance of intensity modulated radiation therapy (IMRT) and volumetric modulated arc therapy (VMAT) quality assurance (QA; further referred to as IMRT QA) is often questioned^1^, ^2^, ^3^, ^4^ due to the lack of sensitivity of the IMRT QA methods. Recent recommendations^5^ and publications^6^ have indicated that reducing the gamma criteria to 3%/2 mm, 10% threshold with an evaluation of where the dosimetric errors occur and their clinical relevance can increase the sensitivity of the QA process. However, there has been little published on the experience and clinical implications of implementing tighter gamma criteria. This work presents the initial clinical implementation of the work by Stambaugh et al.^6^ that motivated changing our IMRT QA practice to use tighter gamma criteria and action thresholds as well as change from a simple pass/fail decision to a more nuanced pass/question/fail workflow. These changes in turn revealed previously unappreciated differences between linear accelerator (LINAC) measurements and treatment planning system (TPS) calculations and the need to improve aspects of our treatment planning calculations to better represent the patient dose for physician review. Detailing these changes and their effect on IMRT QA results will aid clinics in implementing TG‐218.

## 2 MATERIALS AND METHODS

### 2.A Retrospective IMRT QA analysis

One hundred eighty plans, 101 head and neck and 79 prostate VMAT/IMRT (further referred to as IMRT) plans analyzed in the preceding year were collected. All plans had been planned using version 13.7 of the Eclipse TPS (Varian Medical Systems, Palo Alto, CA) using the Anisotropic Analytical Algorithm (AAA) and measured on a Varian Clinac iX (Varian Medical Systems, Palo Alto, CA) equipped with 120‐leaf Millennium MLCs (5 mm leaf width in the central region) or on a Varian TrueBeam equipped with 120‐leaf Millennium MLCs (5 mm leaf width in the central region) which was matched to the Clinac for the 6 and 18 MV beam models. Cumulative dose measurements had been taken using the ArcCHECK helical diode array phantom (Sun Nuclear Corp., Melbourne, FL, USA). All plans had been analyzed during the initial QA with SNC Patient (Sun Nuclear Corp., USA) using the gamma criteria^7^ of 3%/3 mm, global and 10% threshold (The software switch “Apply measurement uncertainty” was on, so the criteria correlates with 4%/3 mm with uncertainty correction off). A passing threshold of 92% had been used for many years, and all these plans had passed. The plans were reanalyzed with the new gamma criteria of 3%/2 mm, global, 10% threshold, and uncertainty corrections off, and the plans were binned into the categories of “pass” (>95% of diodes passing), “questionable” (between 90% and 95%), and “failed” (<90%).^6^ The results using the new criteria were used to obtain an estimate for the number of plans requiring additional dose volume histogram (DVH)‐based analysis using 3DVH software (Sun Nuclear Corp., USA) and therefore the predicted workload increase.

The implementation of the revised IMRT QA analysis was phased. For the first 2 months, 133 IMRT QA measurements made and analyzed with the original criteria and used for the patient‐specific QA documentation while, in parallel, the results with the revised criteria were obtained and tracked. This was done to permit training with the new workflow and to test whether the earlier prediction of the increased workload would be borne out. The distribution of pass, questionable, and fail plans under the new criteria was compared to the predicted distribution using a chi‐square test.

There was an observed increased frequency of problematic plans once all IMRT QA were included in the new analysis method (see Table 1 below). DVH analysis demonstrated that over 50% of the problematic plans were measuring cold in the organs at risk (OAR) and hot in the planning target volume (PTV), which indicated that there was a systematic difference between the TPS and resulting treatment. This motivated a series of investigations and modifications to our processes in order to reduce the effects of systematic calculational artifacts. These are detailed below. Subsequent to these changes, the new criteria were clinically implemented. The distribution of IMRT QA results between “pass,” “questionable,” and “fail” was analyzed for the next 673 plans over 15 months.

**Table 1:** Distributions of pass, questionable, and failed plans for the retrospective analysis of H & N and prostate IMRT quality assurance (QA) measurements, the first 2 months of clinical testing prior to introducing the calculational changes, and the subsequent 15 months of IMRT QA measurements. The gamma criteria are 3%/2 mm, 10% threshold with >95% of points passing being a pass, between 90% and 95% being questionable and below 90% being a fail

	Pass	Questionable	Fail
Predicted (*N* = 180)	135 (75%)	34 (18.9%)	11 (6.1%)
Observed prior to changes (*N* = 133)	71 (53.4%)	47 (35.3%)	15 (11.3%)
Observed after changes (*N* = 674)	582 (86.3%)	88 (13.1%)	4 (0.6%)

### 2.B Calculational changes

#### 2.B.1 TPS calculation parameters

It was found that one of the distributed calculation framework (DCF) settings in Eclipse, which sets the angular resolution for conformal and VMAT calculations, was set to 5 degrees. This caused an apparent ripple in the calculated dose, most prominently for small fields and increasing with distance from the isocenter. This setting was changed to “off” which changed the calculation resolution to the resolution of the control point (2 degrees). The effect is shown in Section 3.

**Figure 1:**
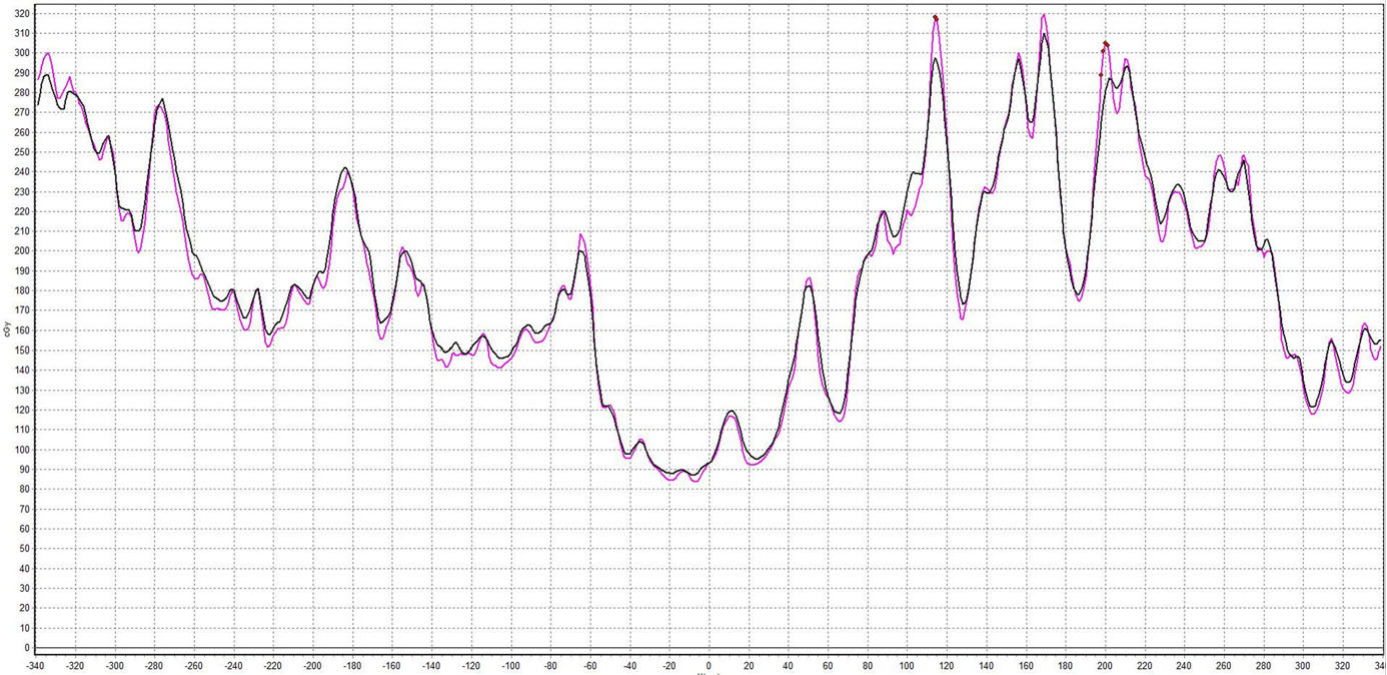
Eclipse calculated 1 mm grid displayed in purple and Eclipse calculated 2.5 mm grid interpolated to 1 mm by SNC displayed in black.

The clinical dose calculation grid size is typically set to 2.5 mm in Eclipse. However, SNC Patient interpolates all plans to a 1.0 mm grid for gamma analysis creating opportunities for misrepresentations of treatment plans as shown in Fig. 1. Now all QA plans are calculated with a dose grid of 1.0 mm to remove any interpolation error.

### 2.B.2 QA phantom calibration

The model for the ArcCHECK phantom within Eclipse was checked to ensure the correct bulk density was assigned and the model was correct as defined by Sun Nuclear. The calibration routine and the dose calibration factor were retaken to ensure that the ArcCHECK was calibrated correctly for each LINAC it was used on. Simple fields (e.g., parallel opposed 10 × 10 cm) were measured and compared to the TPS to ensure that there was agreement between the two for simple cases.

### 2.B.3 DLG & leaf transmission settings

A single beam model is used for the flattened beams of the Varian Clinac and Truebeam accelerators in our clinic due to the fact that the gold beam and representative PDD and Profile data were found to be comparable. Each accelerator was validated against the gold beam data through a series of point dose measurements for open, wedge, and partially blocked MLC fields. Additionally, clinical plans were chosen that represented the planning practices, and further validation was performed and the model was further tuned using the dosimetric leaf gap (DLG) values that allow AAA to model the MLC's. At the time of commissioning, the MLC leaf transmission values were not adjusted. Since the DLG and transmission factors are two adjustable parameters in Eclipse that allow the agreement between the TPS and measurement results to be improved, these settings for the 6 and 18 MV beams were re‐evaluated for clinical plans. Nine clinical VMAT plans were used for the 6 MV beam model and six static IMRT plans were used for the 18 MV beam model. Clinical plans were picked to be representative of our planning practices. For 6 MV, all anatomic sites were covered with a very heavy bias towards VMAT plans with eight of the nine clinical plans being VMAT, which is representative of our practice. Only static IMRT is permitted for 18 MV and is used infrequently in our clinic. Therefore, six plans were representative of our current planning practices. For each plan, four different DLG/transmission combinations were used to recalculate the plans in Eclipse and then compare to the corresponding measurement taken with the ArcCHECK. Gamma analysis was performed in SNC Patient and passing rates were compared to the passing rates obtained with the initial DLG and transmission parameters. These were altered until agreement degraded, giving a range of possible combinations, and then the parameters were refined until optimal agreement was reached.

### 2.B.4 Couch top model values

**Figure 2:**
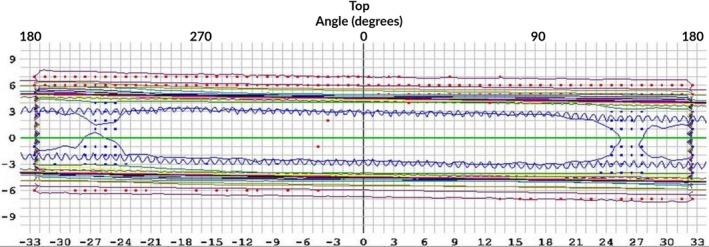
ArcCHECK measurement results of a 360° open arc, including transmission through the couch, compared to the TPS calculation of the same arc. Cold spots occur at ~240° and ~130° which correspond to the thickest portion of the couch.

Due to a discrepancy seen between the TPS and the corresponding ArcCHECK measurement for a 360° open field arc (see Fig. 2 below) and that this same phenomenon was not observed with a 180° arc avoiding the table, it was determined that couch top model being used was inadequate.

**Figure 3:**
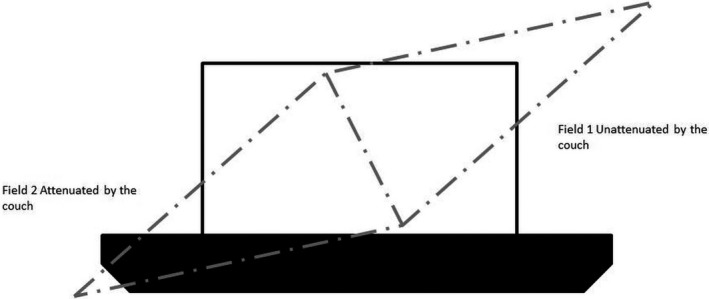
Setup for determining couch top model values using a rectangular solid water phantom as per TG‐176.

AAPM TG‐176^8^ was used to define the attenuation of the couch top through multiple angles and arc lengths. Ideally a cylindrical phantom is available to make complementary angle measurements redundant; however, a cylindrical phantom was unavailable. Therefore, measurements were taken using a slab phantom as described in TG‐176^8^ as the alternate approach and depicted in Fig. 3. Measurements were taken with an ion chamber through the couch into the phantom at angles of 180, 210, 225, and 240 degrees and then 180 degrees opposed (0, 30, 45, and 60) for each angle to get the baseline measurement. An arc of 0–45 degrees and complimentary arc of 180–225 was also measured. The ratios of these readings were taken to be the attenuation of the couch top at various angles.

Eclipse models the couch top via an inner and outer surface. These two variables were iterated to minimize the error for the various angles and arcs measured. The geometry of these models are fixed and hardcoded in Eclipse.

The combined effect of changing the grid size and improving the couch model was tested using 15 single isocenter multiple metastases SRS cases. These were chosen because the effect of grid size would be more important for these plans. Four verification plans were calculated: three were of varying grid sizes and the final plan included the properly modeled couch. (Note that while the SRS treatment beams do not pass through the couch, the IMRT verification measurements do.)

## 3 RESULTS

### 3.A Retrospective IMRT QA analysis

**Figure 4:**
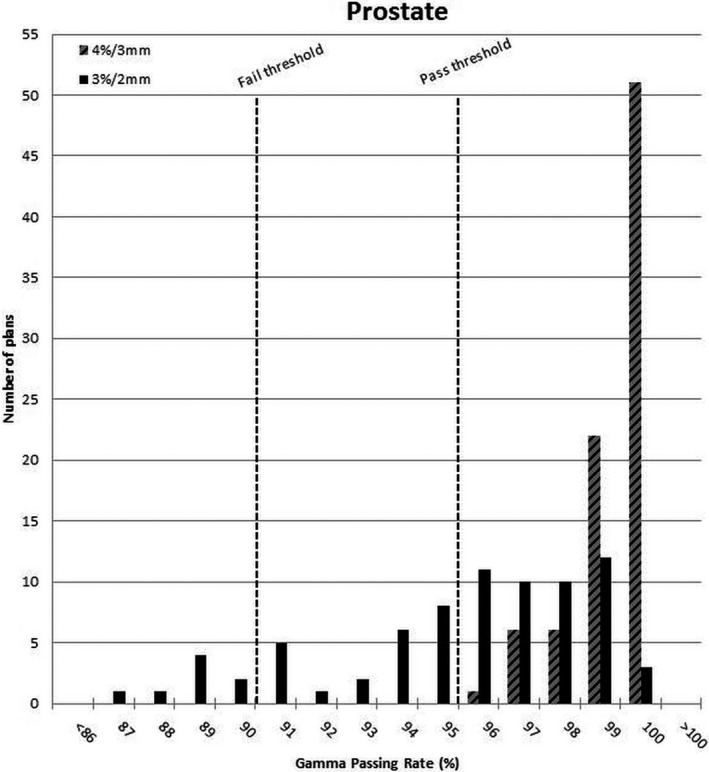
Prostate gamma passing rate results under original and new gamma criteria.

**Figure 5:**
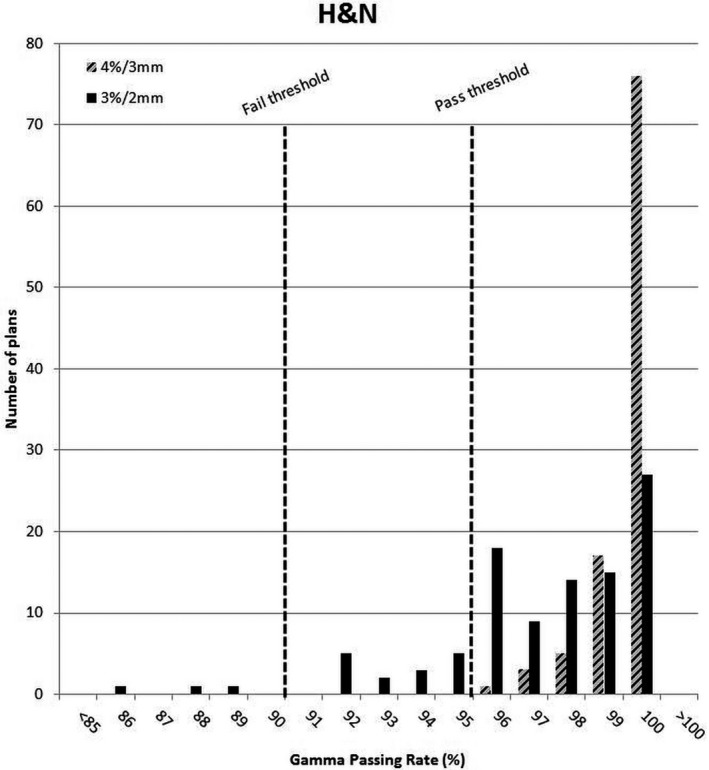
Head and neck gamma passing rate results under original and new gamma criteria.

The retrospective analysis of IMRT QA results showed that all head and neck and prostate plans would have passed the 95% threshold using the original gamma criteria. However, when the new gamma criteria were applied, the gamma passing rate distribution spread out as shown in Figs. 4 and 5.

### 3.A.1 Predicted vs actual distribution of IMRT QA results

Table 1 below shows the distribution of pass, questionable, and failed plans for the retrospective analysis of H & N and prostate IMRT QA measurements, the first 2 months of clinical testing, and the subsequent 15 months of IMRT QA measurements using the tighter gamma criteria.

A chi‐square test of the above distributions showed that the distributions were statistically significantly different (*p* < 0.001).

### 3.B Calculational changes

#### 3.B.1 TPS calculation parameters

##### DCF angular resolution settings

**Figure 6:**
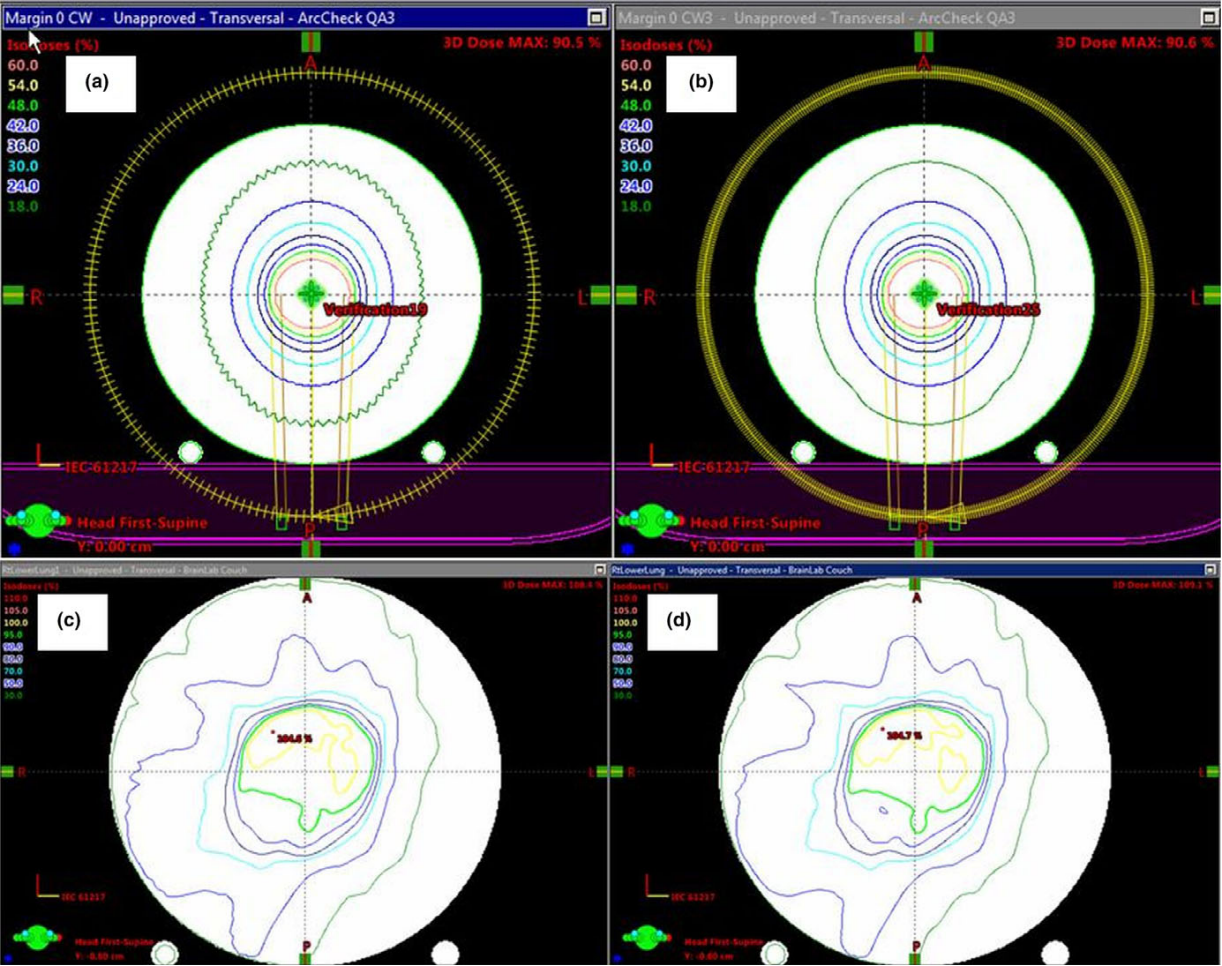
Differences in isodose distributions between the distributed calculation framework (DCF) angular resolution settings (a) 5 cm field, dynamic conformal arc with 5° DCF angular resolution setting (b) 5 cm field, dynamic conformal arc with 0.7° DCF angular resolution setting (c) Lung IMRT plan, DCF angular resolution setting of 5 degrees (d) Lung IMRT plan, DCF angular resolution setting OFF.

**Figure 7:**
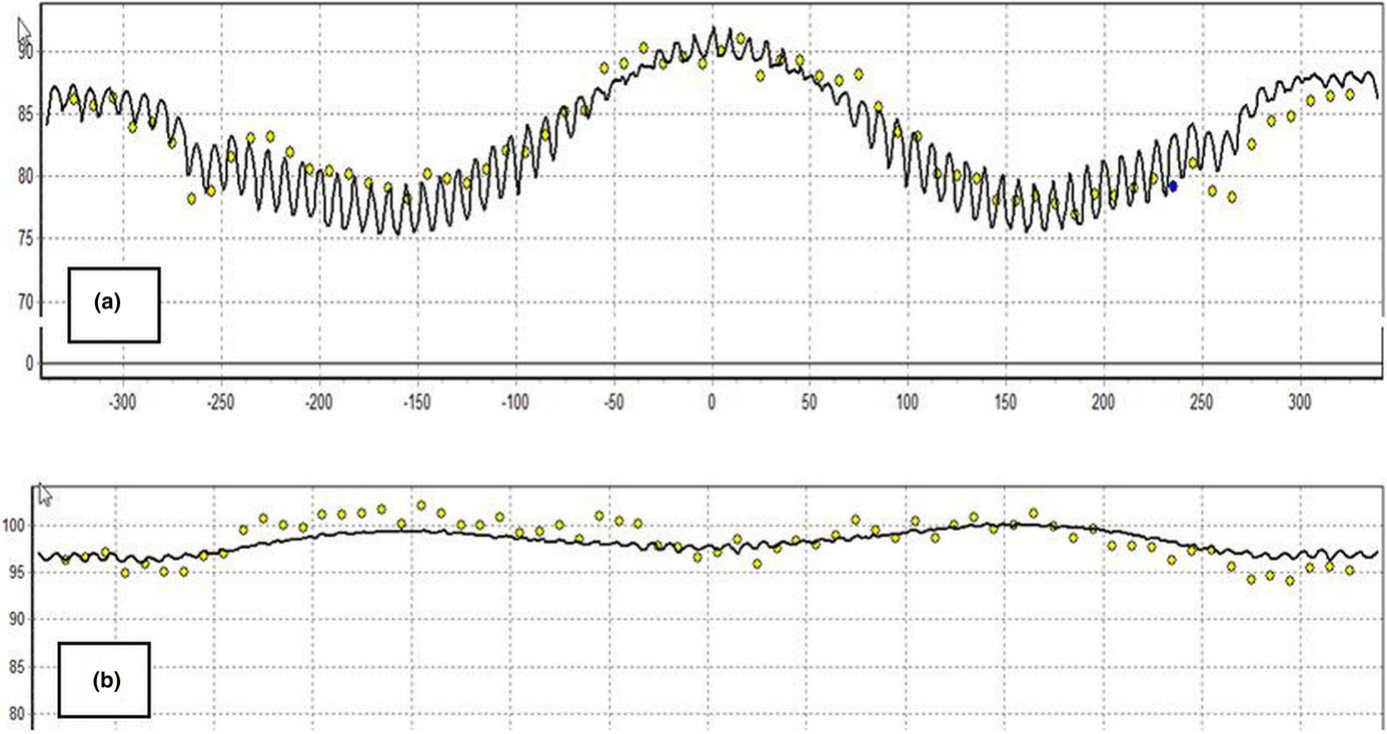
The ArcCHECK measurement results compared to TPS calculations for the same field with (a) distributed calculation framework (DCF) angular resolution setting of 5° and (b) DCF angular resolution setting off.

Figure 6 demonstrates the impact of changing the DCF angular resolution setting from 5 degrees to off, which forces the algorithm to calculate per control point. As can be seen below, using a larger angular resolution of 5 degrees loses small details [Figs. 6(c) and 6(d)] and creates ripples in the dose distribution further from isocenter and closer to the diodes of the ArcCHECK [Figs. 6(a) and 6(b)]. Due to the location of the diodes on the periphery of the ArcCHECK cylinder, this effect creates false variation in the diode reference dose that is used for the measurement comparison. While this has little clinical significance to the target dose, this has a large impact on the measurement results, especially for small field plans as can be seen in Fig. 7. In this case, the 3%/2 mm passing rate for the plan increased from 90.1% to 96.8% by changing the DCF angular resolution setting. The gamma results for larger field plans also were impacted, although not as drastically (~2% increase) as small fields as can be seen in Table 2.

**Table 2:** Gamma passing rate comparison for five sites with large fields with distributed calculation framework (DCF) setting of 5° and OFF

Site	3%/2 mm DCF 5°	3%/2 mm DCF OFF	Difference (%)
H&N	90.4	93.6	3.2
H&N	95.8	97.6	1.8
Abdomen	94.1	97.0	2.9
Lung and mediastinum	93.5	94.8	1.3
Prostate bed + nodes	89.1	91.1	1.9

##### Couch modeling and dose grid results

The original couch models demonstrated up to a 4.2% difference between the TPS and the corresponding measurement for select beam directions. After optimizing the surface and interior HU values for the couch in Eclipse, the error between the TPS plan and measurement decreased to less than 0.5%.

Table 3 demonstrates the average gamma passing rate of all 15 SRS plans along with their standard deviation as the grid increment was reduced and, finally, the improved couch model assigned.

**Table 3:** Average gamma passing rate of SRS VMAT plans calculated at varying grid sizes and couch model

Verification plan	Results
2.5 mm grid	92 ± 5%
1.25 mm grid	96 ± 3%
1.0 mm grid	97 ± 2%
1.0 mm grid with improved couch model	98.2 ± 1.3%

#### 3.B.2 DLG & leaf transmission settings

After iterating through four different DLG/transmission factors, it was found that for 6 MV, changing the DLG from 0.24 cm to 0.21 cm and changing the transmission factor from 0.015 to 0.017, increased the gamma passing rates for all plans measured. This was true for both the Clinac and TrueBeam and decreased the maximum difference between gamma passing rates for the two machines from 6% to 4.8% (Table 4).

**Table 4:** Comparison of Clinac and TrueBeam increase in gamma passing rates and agreement before and after the change in DLG and transmission values

	Ave % increase in passing rate		Ave % Diff between Clinac/TrueBeam		Max % diff between Clinac/TrueBeam	
	Clinac	TrueBeam	Original beam model	New beam model	Original beam model	New beam model
6 MV	1.8	2.0	0.9	1.2	6.0	4.8
18 MV	1.8	−0.3	2.8	0.8	7.6	3.5

For 18 MV, it was found that changing the DLG from 0.1817 to 0.2 cm and keeping the transmission factor at 0.0152 gave the most optimal results. This increased the passing rate for all plans measured on the Clinac. When the plans were measured on the TrueBeam, half saw an increase in gamma passing rate while the other half saw a slight decrease in gamma passing rate. However, the maximum percent gamma passing rate difference between the Clinac and TrueBeam decreased from 7.6% to 3.5% (Table 4).

## 4 DISCUSSION

This work confirmed the expectation that tightening IMRT QA tolerance levels, as recommended by AAPM TG‐218^5^ and validated by Stambaugh,^6^ would result in many more QA results being deemed questionable or failing. Indeed, prior to implementing the revised QA parameters, it was exceedingly rare in our institution for a plan to fail IMRT QA. This work also demonstrated that looking at IMRT QA results more critically revealed problems with our calculation processes that had previously been unappreciated and that a comprehensive evaluation of QA systems as a whole is necessary. This is consistent with the recommendations of TG‐218^5^ on how to respond to failing or marginal IMRT QA results.

None of the calculational improvements detailed here were individually very large. However, the cumulative effect was to dramatically reduce the frequency of apparently questionable or failing plans. As seen in Table 1, implementing these improvements decreased the frequency of questionable plans from 35% to 13% and of failing plans from 11% to less than 1%. Some of those changes affect patient and verification plans (e.g., IMRT modeling parameters) and some affect primarily the verification plans (e.g., angular resolution of the calculation). Improving the accuracy of the patient plans is inherently valuable, as it provides physicians with more accurate information when evaluating plans, and improving the accuracy of the verification plans has important practical implications. Responding to marginal or failing QA results involves considerable work. Eliminating unnecessary work engendered by calculational artifacts makes implementing the recommendations of TG‐218^5^ much more palatable in a busy clinic.

Even after these improvements, about 14% of our IMRT plans do not cleanly pass IMRT QA. We are still in the process of learning from DVH analysis of the questionable and failing plans if further systematic improvements to our treatment planning processes are warranted or if we have reached the limit based on the changeable parameters available within Eclipse. One known issue is that we have a beam model that is optimized to minimize differences between the Varian Clinac and Truebeam. In the next year we will have completely transitioned to the newer machines and will adjust the models accordingly.

## 5 CONCLUSIONS

Tightening IMRT QA tolerances revealed the need to improve several elements of our IMRT and VMAT calculations. As a consequence, the accuracy of our treatment planning has improved, and the frequency of finding marginal or failing IMRT QA results, while much larger than before the tightening, remains within our practical ability to respond.

## CONFLICTS OF INTEREST

The authors have no relevant conflicts of interest to disclose.
